# The role of point-of-care ultrasound (POCUS) imaging in clinical outcomes during cardiac arrest: a systematic review

**DOI:** 10.1186/s13089-023-00346-1

**Published:** 2024-01-24

**Authors:** Hany A. Zaki, Haris Iftikhar, Eman E. Shaban, Mavia Najam, Baha Hamdi Alkahlout, Nabil Shallik, Wael Elnabawy, Kaleem Basharat, Aftab Mohammad Azad

**Affiliations:** 1 Emergency Medicine, Hamad General Hospital, P.O. Box 3050, Doha, Qatar; 2 Cardiology, Al Jufairi Diagnosis and Treatment, Doha, Qatar; 3 Department of Medical Education, Hamad Medical Corporation, Doha, Qatar; 4 Anesthesia Department, IT Deputy Chair, HMC, Doha, Qatar

## Abstract

**Background:**

Cardiac arrest in hospital and out-of-hospital settings is associated with high mortality rates. Therefore, a bedside test that can predict resuscitation outcomes of cardiac arrest patients is of great value. Point-of-care ultrasound (POCUS) has the potential to be used as an effective diagnostic and prognostic tool during cardiac arrest, particularly in observing the presence or absence of cardiac activity. However, it is highly susceptible to “self-fulfilling prophecy” and is associated with prolonged cardiopulmonary resuscitation (CPR), which negatively impacts the survival rates of cardiac arrest patients. As a result, the current systematic review was created to assess the role of POCUS in predicting the clinical outcomes associated with out-of-hospital and in-hospital cardiac arrests.

**Methods:**

The search for scientific articles related to our study was done either through an electronic database search (i.e., PubMed, Medline, ScienceDirect, Embase, and Google Scholar) or manually going through the reference list of the relevant articles. A quality appraisal was also carried out with the Quality Assessment of Diagnostic Accuracy Studies tool (QUADAS-2), and the prognostic test performance (sensitivity and sensitivity) was tabulated.

**Results:**

The search criteria yielded 3984 articles related to our topic, of which only 22 were eligible for inclusion. After reviewing the literature, we noticed a wide variation in the definition of cardiac activity, and the statistical heterogeneity was high; therefore, we could not carry out meta-analyses. The tabulated clinical outcomes based on initial cardiac rhythm and definitions of cardiac activity showed highly inconsistent results.

**Conclusion:**

POCUS has the potential to provide valuable information on the management of cardiac arrest patients; however, it should not be used as the sole predictor for the termination of resuscitation efforts.

## Introduction

Cardiac arrest, which is characterized by an abrupt loss of cardiac output and contractility, has a very high death rate in both hospital and out-of-hospital settings. Global statistics show that the incidence of out-of-hospital cardiac arrests (OHCA) is approximately 55 per 100,000 adults [1]. Similarly, statistics provided by the American Heart Association show that about 356,000 out-of-hospital cardiac arrests (OHCA) are witnessed in the United States annually, of which 90% are fatal [2]. Therefore, a bedside test that can predict resuscitation outcomes of cardiac arrest patients is of great value.

Point-of-care ultrasound (POCUS) provides emergency physicians with a diagnostic and prognostic tool, especially in cardiac arrest, where physical examination is not always accurate [3, 4]. Historically, POCUS in cardiac arrest was used to identify reversible causes such as cardiac tamponade and right heart strain, which is suggestive of massive pulmonary embolus [5, 6]. However, research shows that it has the potential to be used as an effective diagnostic and prognostic tool during cardiac arrest, particularly in observing the presence or absence of cardiac activity [7]. Despite the literature associating cardiac activity with return of spontaneous circulation (ROSC), POCUS is highly susceptible to bias from “self-fulfilling prophecy” as most physicians are usually not blinded to the outcomes of this test. Furthermore, research suggests that POCUS prolongs Cardiopulmonary resuscitation (CPR), which negatively impacts the survival rates of cardiac arrest patients [8, 9]. Therefore, we conducted an up-to-date prognostic factor systematic review on POCUS during cardiac arrest.

## Methods

### Protocol and registration

We conducted this systematic review in accordance with the Cochrane Collaboration guiding principles. Our results were reported as per the PRISMA (Preferred Reporting Items for Systematic Review and Meta-Analyses) guiding principles [10].

### Search methods

An extensive literature search was carried out on PubMed, Medline, ScienceDirect, Embase, and Google Scholar for all publications between 2000 and December 2022 using the following strategy: (“Point-of-care ultrasound” OR “bedside ultrasound” OR “ultrasonography” OR “ultrasound” OR “POCUS” OR “bedside cardiac ultrasound” OR “echocardiography”) AND (“Cardiac Arrest” OR “Sudden cardiac arrest” OR “Heart attack”) AND (“survival” OR “mortality” OR “return of spontaneous circulation” OR “Cardiac activity”). In addition, reference lists of potential studies were scrutinized for more studies. Grey literature and exact or close duplicates were also eliminated as they would interfere with the scientific purpose of the present research.

### Selection criteria, outcomes and definitions

Two review authors used the PICOST (Population, Intervention, Comparator, Outcome, Study design, Time frame) framework to formulate study questions used to include articles in the current review. The proposed framework was as follows; adult patients with IHCA or OHCA (P); point-of-care ultrasound or echocardiography during cardiopulmonary resuscitation (CPR) (I); absence of finding or different finding on POCUS during CPR (C); prognostic clinical outcomes, i.e., return of spontaneous circulation (ROSC), survival to hospital admission (SHA) and survival to hospital discharge (SHD) (O); human randomized controlled studies and observational studies (prospective, cross-sectional and retrospective) (S); studies published from the year 2000 and written in English (T). Studies that included pediatric patients, animal studies, letters to the editor, case reports and series, systematic reviews and meta-analysis, guidelines, and abstracts without full articles were also excluded.

The main outcomes analyzed in this review article were: ROSC, SHD and SHA. These outcomes were analyzed according to the initial cardiac arrest rhythm, definition of cardiac activity and level of POCUS training.

The definition of “cardiac activity” is very heterogeneous as it varies from study to study; however, in the current review it was defined a priori as any visible movement of the myocardium or valvular contraction. In addition, the level of training was classified as either experienced or inexperienced. Inexperience referred to POCUS operators who had to undergo hands-on and theoretical training before carrying out POCUS examinations, while experienced referred to operators who had undergone the training and had at least 2 years of experience with POCUS.

### Quality assessment

The risk of bias assessment was independently carried out by two reviewers using the Quality Assessment of Diagnostic Accuracy Studies (QUADAS-2) tool embedded in the Review Manager software (RevMan 5.4.1) (Kappa 0.81). This tool generally comprises 4 bias assessment domains, including participants/patient selection, index test, reference standard, and flow and timing, and 3 applicability domains, including patient selection, index test, and reference standard.

### Data extraction

The process of extracting relevant data was entrusted to two reviewers, who collated and summarized the pertinent study data, demographic data, initial cardiac rhythm, cardiac arrest setting, POCUS operator, and study outcomes (Table 1). The reviewers discussed any disagreements that occurred throughout the data extraction process or sought advice from a third reviewer who functioned as the arbiter.

**Table 1:** Study characteristics

Author ID	Study design	Country	Cardiac arrest setting	Patients’ characteristics					Operator	POCUS setting	Outcomes
				Sample (*n*)	M/F	Initial rhythm					
						Asystole	PEA	VF/VT			
Aichinger et al. [11]	Prospective observational study	Austria	OHCA	42	30/12	20	11	11	EP	Pre-hospital	SHA
Atkinson et al. [12]	Retrospective observational study	Canada	OHCA	223	149/74	NR	NR	NR	EP	ED	SHA, SHD and ROSC
Blaivas et al. [13]	Prospective observational study	United States	OHCA	169	NR	65	38	66	EP	ED	SHA
Bolvardi et al. [14]	Prospective observational study	Iran	IHCA or OHCA	159	83/76	79	50	30	EP	ED	ROSC
Breitkreutz et al. [15]	Prospective observational study	Germany	OHCA	230	141/84	38	22	35	EP	Pre-hospital	SHA
Cebicci et al. [16]	Retrospective observational study	Turkey	IHCA or OHCA	410	132/278	290	75	45	EP	ED	SHA and ROSC
Chardoli et al. [17]	Prospective observational study	Iran	OHCA	100	56/44	NR	100	NR	EP	ED	ROSC
Chua et al. [18]	Prospective observational study	Singapore	OHCA	104	71/33	47	33	17	Senior resident	ED	SHA and SHD
Cureton et al. [19]	Retrospective observational study	United States	OHCA	318	NR	NR	318	NR	EP	ED	SHA
Flato et al. [20]	Prospective observational cohort study	Brazil	IHCA	49	27/22	17	32	NR	ICU specialists	ICU	SHD and ROSC
Gaspari et al. [21]	Prospective observational study	United States and Canada	IHCA or OHCA	793	492/301	379	414	NR	EP	ED	SHA, SHD and ROSC
Hayhurst et al. [22]	Prospective observational study	United Kingdom	OHCA	50	NR	20	23	6	EP	ED	SHA and ROSC
Kim et al. [23]	Prospective observational study	Republic of Korea	OHCA	48	34/14	39	8	1	Senior residents and EP	ED	ROSC
Lien et al. [24]	Prospective observational study	Taiwan	OHCA	177	111/66	82	64	31	EP	ED	ROSC and SHD
Masoumi et al. [25]	Cross-sectional study	Iran	IHCA or OHCA	151	115/36	89	62	NR	EP	ED	SHA, SHD, and ROSC
Ozen et al. [26]	Prospective single-center observational Study	Turkey	IHCA or OHCA	129	83/46	NR	NR	30	Senior emergency residents	ED	SHA and ROSC
Salen et al. [27]	Prospective observational study	United States	IHCA or OHCA	102	NR	36	55	11	EP, residents, and attendings	ED	SHA
Salen et al. [28]	Prospective, multicenter observational trial	United States	OHCA	70	43/27	36	34	NR	EP	ED	SHD and ROSC
Tayal et al. [29]	Prospective observational study	United States	OHCA	20	12/8	NR	NR	NR	EP	ED	SHD and ROSC
Thandar et al. [30]	Prospective cohort study	India	OHCA or IHCA	84	57/37	56	28	NR	EP	ED	SHA, SHD and ROSC
Tomruk et al. [31]	Prospective observational study	Turkey	OHCA or IHCA	149	97/52	77	64	8	EP	ED	ROSC
Zengin et al. [32]	Prospective observational study	Turkey	IHCA or OHCA	179	104/75	108	42	5	Senior doctors	ED	SHD

*EP* emergency physicians, *POCUS* point-of-care ultrasound, *SHA* survival to hospital admission, *SHD* survival to hospital discharge, *ROSC* return of spontaneous circulation, *PEA* pulseless electrical activity, *VF* ventricular fibrillation, *VT* ventricular tachycardia, *NR* not reported, *ED* Emergency Department

### Data synthesis

The present systematic review was constructed for prognosis rather than diagnostic test accuracy; therefore, the specificity and sensitivity of cardiac activity were used to predict the resuscitation outcomes (i.e., ROSC, SHA, and SHD) of cardiac arrest using POCUS. Initially, we planned to carry out meta-analyses of the clinical outcomes; however, after analyzing the studies we found that the pooled data had substantial statistical heterogeneity (I2 > 50%), thus all meta-analyses were eliminated. In the ROSC sensitivity and specificity analysis, we defined true positive as the number of individuals achieving ROSC with cardiac activity on POCUS, while false negative defined patients achieving ROSC without cardiac activity. On the other hand, true negative defined non-ROSC without cardiac activity, and false positive defined non-ROSC with cardiac activity. Similarly, in the analysis of SHA and SHD, true positives, false positives, true negatives, and false negatives referred to patients with cardiac motion and surviving to admission or discharge, patients with cardiac activity but not surviving to admission or discharge, patients without cardiac activity and not surviving to admission or discharge, and patients without cardiac activity but surviving to admission or discharge, respectively.

## Results

### Study selection

**Fig. 1:**
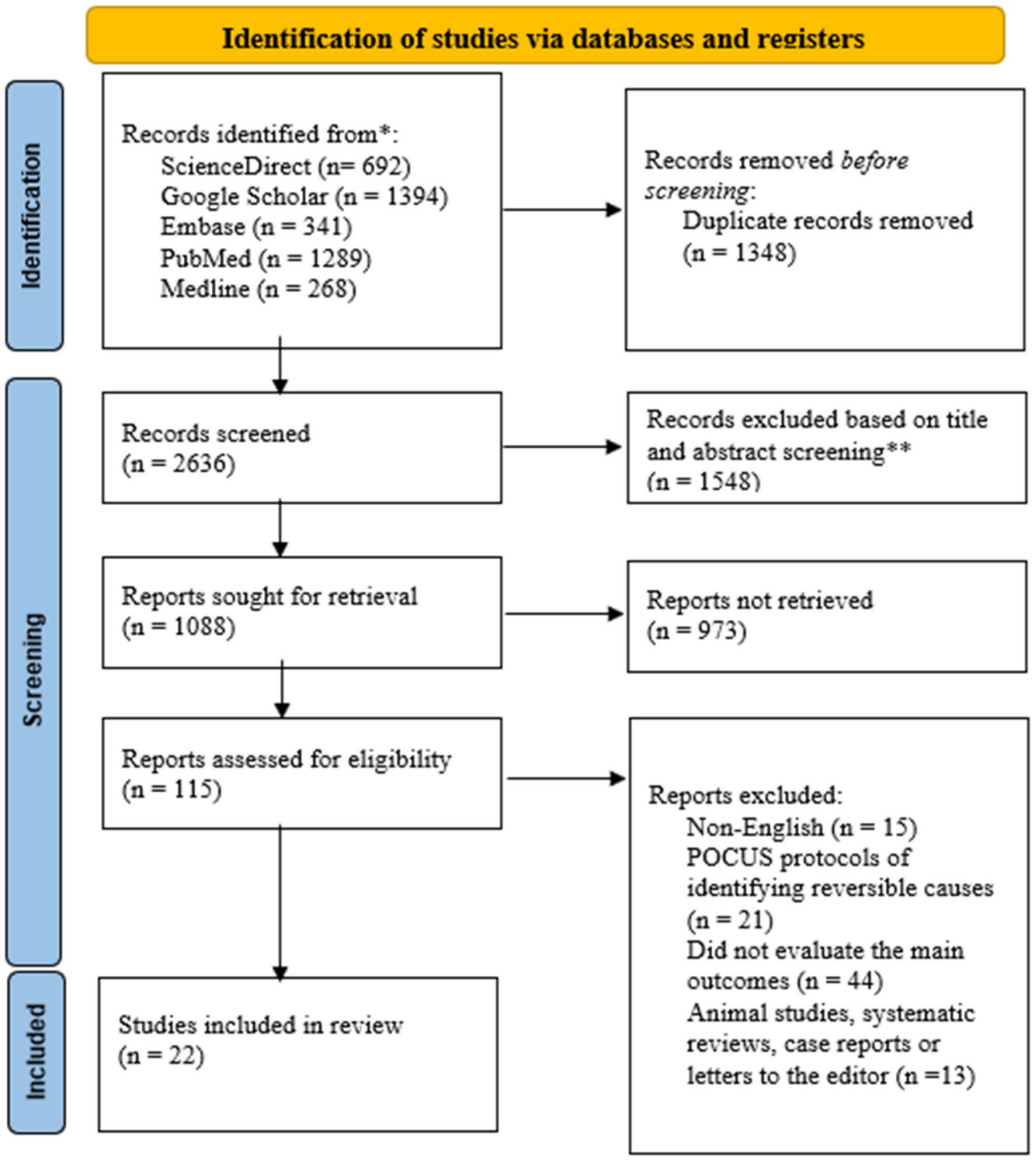
PRISMA diagram for literature selection

The thorough database search accumulated 3984 articles of which 2896 were excluded based on the duplicate check and screening criteria. After employing the eligibility criteria on 115 articles, 93 were excluded and 22 scientific journals were included for review. The full selection criteria are outlined in the PRISMA diagram below (Fig. 1).

### Bias assessment

**Fig. 2:**
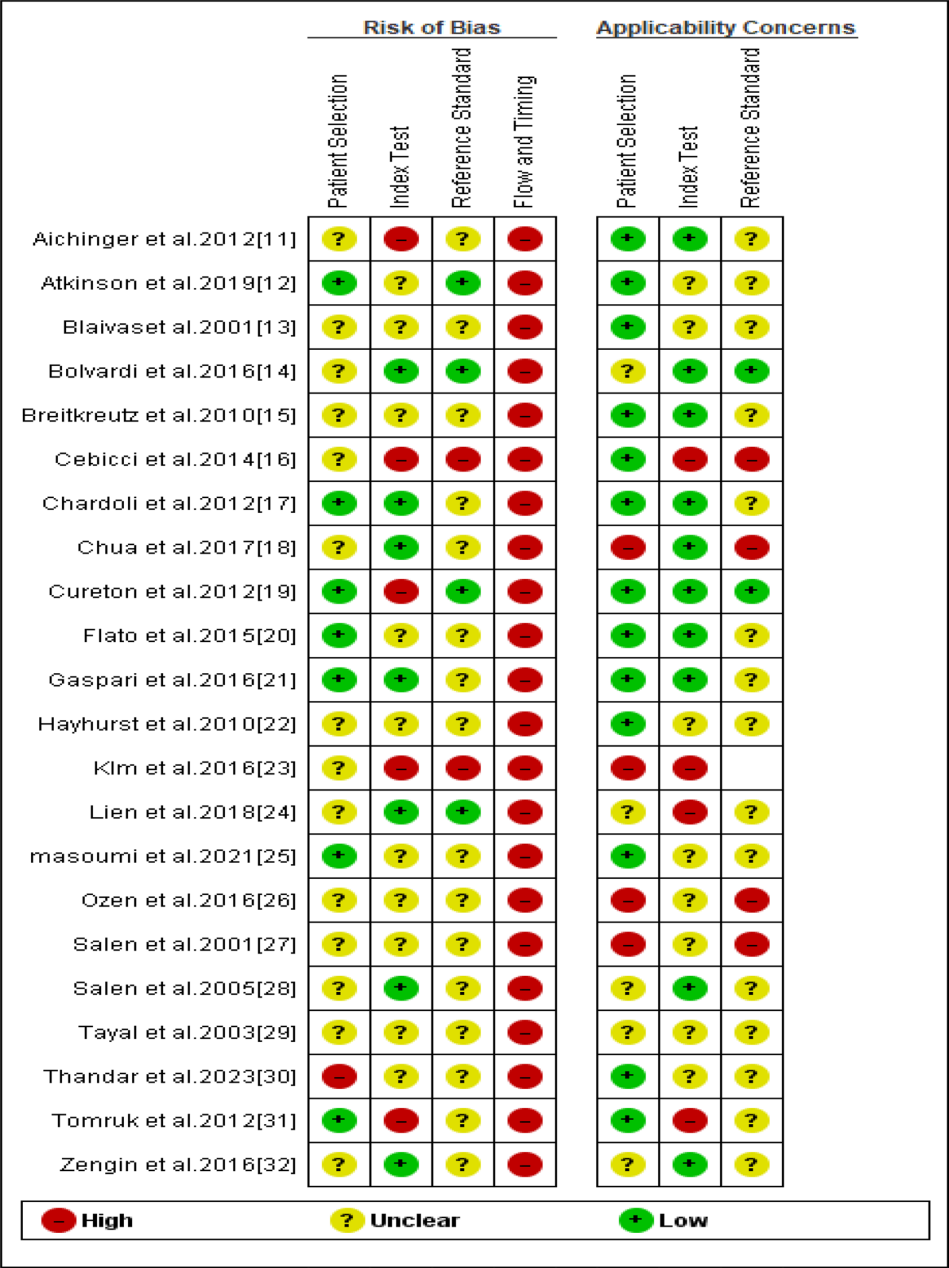
QUADAS-2 bias assessment summary

The results of the bias assessment performed using the QUADAS-2 tool are provided in Fig. 2 below. Our assessment revealed that all studies had a high risk of flow and timing bias since physicians were not blinded to the POCUS results. This lack of blinding may have influenced the decision to terminate resuscitation efforts, creating a self-fulfilling prophecy. Moreover, our assessment revealed that some studies had patient selection concerns due to patient exclusions, convenience sampling, and single-center designs. Unclear and high risk of bias was also seen in the index test due to the lack of pre-defined cut-offs and the fact that some physicians had access to electrocardiogram results before performing the POCUS exams. Overall, the risk of bias across the studies was high, thus, lowering the certainty of evidence from these studies (Table 1).

### Does the presence of cardiac activity on pre-hospital POCUS predict resuscitation outcomes of cardiac arrest patients?

Two observational studies [11, 15] of 272 OHCA patients reported the role of pre-hospital POCUS in predicting resuscitation outcomes of cardiac arrest patients (Table 2). The outcomes from these studies were further stratified according to the initial cardiac rhythm and the definition of cardiac activity. The review of data from these studies demonstrated a sensitivity range of 0.95–1.00 and a specificity range of 0.41–0.70 for SHA in patients presenting PEA as the initial rhythm and ranges of sensitivity (0.50–0.69) and specificity (0.83–1.00) in asystole patients. On the other hand, only one study reported resuscitation outcomes for patients with VF/VT rhythms, of which cardiac activity predicted SHA with a 1.00 (95% CI 0.15–1.00) sensitivity and 0.67 (95% CI 0.30–0.93) specificity [11].

When the outcomes were stratified according to the definition of cardiac activity, one observational study with unspecified cardiac activity reported a sensitivity of 0.86 (95% CI 0.70–0.94) and specificity of 0.60 (95% CI 0.47–0.725) for SHA [15]. The other study where cardiac activity was defined as any movement of the myocardium reported sensitivity (0.80; 95% CI 0.31–0.97) and specificity (0.84; 95% CI 0.68–0.93) for SHA [11].

Considering the findings in these studies, it is evident that cardiac activity on pre-hospital POCUS has an inconsistent prognostic value. Therefore, for patients with OHCA, resuscitation is still the main priority and should be managed according to the advanced life support (ALS) guidelines [33].

### Does the presence of cardiac activity on in-hospital (ICU & ED) POCUS predict resuscitation outcomes of cardiac arrest patients?

In patients with PEA as the initial cardiac arrest rhythm, the presence of cardiac activity predicts the possibility of ROSC with a sensitivity range of 0.43–1.00 and a specificity range of 0.33–0.93, while it predicts the likelihood of SHA with a sensitivity range of 0.67–1.00 and specificity range of 0.50–0.93 [13, 16–19, 23, 27, 31]. On the other hand, for patients with Asystole as the initial rhythm, the presence of cardiac activity predicts the possibility of ROSC with a sensitivity range of 0.0–0.11 and specificity range of 0.97–1.00 while it predicts the likelihood of SHA with a sensitivity range (0.0–1.00) and specificity range (0.85–1.00) [13, 16, 18, 23, 27, 28, 31]. Similarly, the presence of cardiac activity for patients with VF/VT as the initial rhythm can estimate the possibility of ROSC with ranges of sensitivity (0.0–1.00) and specificity (0.0–1.00) and the possibility of SHA with a range of sensitivity (0.94–1.00) and specificity (0.50–1.00) [13, 16, 23, 27, 31] (Table 2).

After stratifying the outcomes based on the definition of cardiac activity, we observed that the presence of unspecified cardiac activity had sensitivity ranges of 0.62–0.73 and 0.72–0.94 and specificity ranges of 0.92–0.98 and 0.60–0.98 for ROSC and SHD, respectively [14–16, 18, 24]. One observational study also reported that presence of unspecified cardiac activity had a sensitivity of 0.48 (95% CI 0.28–0.69) and specificity of 0.77 (95% CI 0.69–0.83) for SHD [24] (Table 2).

On the other hand, 5 observational studies [11, 13, 21, 30, 32] with 1267 OHCA and IHCA patients defined cardiac activity as the presence of any movement of the myocardium. Outcomes from two of these studies reported a sensitivity range of 0.52–0.64 and a specificity range of 0.78–0.95 for ROSC [21, 30]. Conversely, 4 of the 5 observational studies reported ranges of sensitivity (0.31–0.98) and specificity (0.73–0.95) for SHA [11, 13, 21, 30], while 3 reported ranges of sensitivity (0.40–0.77) and specificity (0.68–0.83) for SHD [21, 30, 32] (Table 2).

In addition, 8 observational studies [17, 22, 23, 25, 26, 28, 29, 31] with 717 IHCA and OHCA patients defined cardiac activity as any atrial, valvular, or ventricular motion. 7 of these studies reported sensitivity ranges of 0.25–0.95, and specificity ranges of 0.33–0.95 for ROSC [17, 22, 23, 25, 26, 28, 31], while other 3 articles reported ranges of sensitivity (0.75–0.98) and specificity (0.60–0.79) for SHA [22, 25, 26]. Conversely, two studies reported sensitivity ranges of 0.85 to 1.00 and specificity ranges of 0.62–0.74 for SHD[25, 29] (Table 2).

Finally, organized cardiac activity was reported in 3 observational studies [12, 19, 20]. Outcomes from two of these articles showed that organized cardiac activity predicted the possibility of ROSC with a sensitivity range of 0.34 to 0.53 and a specificity range of 0.38–0.96 [12, 20]. Similarly, organized cardiac activity predicted the likelihood of SHA and SHD with sensitivity ranges of 0.39–0.86 and 0.67–1.00 and specificity ranges of 0.38–0.96 and 0.91, respectively (Table 2).

### Does the level of POCUS training influence the ability to predict clinical outcomes of cardiac arrest?

The current review shows that in cardiac arrest patients where inexperienced sonographers perform POCUS exams, cardiac activity predicts the possibility of ROSC with a sensitivity range of 0.26–0.62 and a specificity range of 0.89–0.98 [24, 30, 31], while it predicts the possibility of SHA with a sensitivity range of 0.31–0.98 and specificity range of 0.67–0.91 [11, 13, 18, 28, 30]. Additionally, four observational studies reported a sensitivity ranges of 0.40–1.00 and specificity ranges of 0.62–0.83 for SHD [24, 29, 30, 32] (Table 2).

On the other hand, cardiac activity observed by experienced sonographers, had a predictive sensitivity range of 0.25–0.95 and a specificity range of 0.70–0.96 for ROSC [12, 21–23, 25, 26] and sensitivity range of 0.39–0.94 and specificity range of 0.64 to 0.98 for SHA [12, 16, 19, 21, 22, 25]. Furthermore, outcomes from 4 studies reported ranges of sensitivity (0.67–1.00) and specificity (0.51–0.89) for SHD [12, 20, 21, 25] (Table 2).

Considering the evidence in these studies, POCUS performed by relatively inexperienced physicians (i.e., those with less than 2 year experience) seems to have a similar prognostic value as that performed by experienced physicians. However, more high-quality randomized trials are required to support this finding. Furthermore, we noticed a wide variation in sensitivity and specificity values when POCUS was performed by experienced sonographers. Although, the definitive cause of this variation is not well known, it can be attributed to factors such as different definitions of cardiac activity, the initial cardiac rhythm, and number of echocardiography findings.

## Discussion

The current study was designed to evaluate the ability of POCUS to predict resuscitation outcomes in adult cardiac arrest patients in any setting. Unfortunately, we could not pool clinical outcomes in meta-analyses due to the high risk of bias and statistical heterogeneity between studies.

The main goal of using POCUS in cardiac arrest is to improve resuscitation outcomes by identifying cardiac activity [34]. However, after reviewing articles in the current study, we noticed a wide variation in the definition of cardiac activity. This finding is consistent with a previous systematic review by the Advanced Life Support Task Force of the International Liaison Committee on resuscitation [35]. Moreover, the present study has shown that irrespective of the POCUS setting and definition of cardiac motion, the sensitivity and specificity values are highly inconsistent, with values as low as 25% [23] and 33% [20] recorded in patients presenting any atrial, valvular, or ventricular movement. This evidence suggests that the presence or absence of cardiac activity is insufficient to inform the decision to terminate resuscitation efforts. Therefore, resuscitation efforts should be continued until they prove futile rather than terminating based on the initial sonographic findings. Moreover, we would recommend that a uniform definition of cardiac activity be generated to assist in interpreting future outcomes.

Although the current study implied that cardiac motion does not inform the decision to terminate resuscitation efforts, there is a high risk of cardiac motion on POCUS being used as a self-fulfilling prophecy. A recent questionnaire about termination of resuscitation revealed that about 19% of physicians and 40% of nurses were comfortable with terminating resuscitation efforts after observing cardiac standstill on the echocardiography [36]. However, it is worth noting that even patients with cardiac standstill on the initial ultrasonographic findings may gain cardiac activity after some time. For instance, Gaspari and colleagues reported that the rate of SHD was approximately 3 to 4 times with cardiac activity on initial ultrasonographic findings. However, patients without cardiac activity had longer resuscitation attempts, of which 11% regained cardiac activity during the resuscitation attempts meaning that the sonographic findings are not static [21]. Therefore, cardiac sonographic findings must be cautiously interpreted [37, 38].

Successful resuscitation of patients with PEA or Asystole requires considerable time and effort. Therefore, POCUS is used to identify cardiac motion in these patients and improve resuscitation outcomes. Our review suggests that in patients with PEA or VT/VF as the initial rhythm, cardiac activity tends to have higher sensitivity for predicting ROSC and SHA compared to patients with Asystole. However, the evidence provided in these studies has a high risk of bias; thus, the certainty of the evidence is subjective. In addition, POCUS can be used to identify reversible causes of PEA and asystole, such as hypovolemia, pulmonary embolism, and pericardial effusion. However, detecting pulmonary embolism during resuscitation is modest at best since the right ventricle is usually dilated [37, 38].

POCUS is also a useful tool in differentiating between true and pseudo-PEA. Pseudo-PEA is described as the presence of myocardial electrical activity without a detectable pulse but with coordinated cardiac activity, while true PEA usually refers to the condition where a patient has myocardial electrical activity without a palpable pulse and cardiac activity [39]. Tomruk and colleagues reported that sonography identified 34.4% cases of pseudo-PEA, of which 68.2% were successfully resuscitated. The study also shows that true PEA was detected in 42 out of 64 patients, of which only 20 went on to have successful resuscitation. Similarly, Breitkreutz et al. [15] reported that bedside ultrasound could detect pseudo-PEA in 38 patients, of which 21 survived until hospital admission while 17 died on the scene. True PEA was also diagnosed in 13 patients, of which only one survived to hospital admission while 12 died on the scene. This distinction between true and pseudo-PEA is important because standard cardiac arrest treatments may result in harmful outcomes among patients with pseudo-PEA.

Interestingly, evidence shows that POCUS can be used to make an actual rhythm diagnosis during resuscitation. Thandar and colleagues reported that three patients initially assessed to be in asystole rhythm were diagnosed with ventricular fibrillation after sonographic exams [30]. This accurate diagnosis was essential in making critical decisions and prompting defibrillation. Similarly, two previous case reports reported that VF mimicking asystole was only diagnosed using ultrasound [40, 41]. This misdiagnosis can be explained by the fact that during resuscitation, electrocardiogram leads may be displaced, causing the monitor to show an asystole rhythm.

Research shows that POCUS carried out by trained physicians allows for better evaluation of quality compressions and quick diagnosis of reversible causes of cardiac arrest [42]. However, there is no evidence to suggest that the level of training might affect resuscitation outcomes of cardiac arrest. Our review found that POCUS carried out by relatively inexperienced physicians has almost similar sensitivity and specificity for predicting resuscitation outcomes as POCUS performed by experienced sonographers. This means that physicians with brief and specific POCUS training can accurately identify cardiac motion during resuscitation. However, more high-quality randomized studies are required to support our findings.

Although the current review only evaluated the role of POCUS in clinical outcomes (SHA, ROSC, and SHD), evidence suggests that POCUS can influence the resuscitation time and the intervention used on cardiac arrest patients. Atkinson and colleagues reported longer resuscitation durations in patients showing cardiac activity on POCUS than patients without cardiac activity (27.33 min (95% CI 17.7–37.0) vs. 11.51 min (95% CI 10.2–12. 8), respectively) [12]. Furthermore, patients who did not undergo the POCUS exam had a significantly lower resuscitation duration (14.36 min; 95% CI 9.89–18.8; *p* = 0.001). This increased resuscitation duration among patients with cardiac activity suggests that emergency physicians and team provided increased resuscitation efforts when cardiac activity was observed and stopped resuscitation earlier for patients not undergoing POCUS or in those without evidence of cardiac activity. This finding in addition to improved ROSC, SHA and SHD rates in that study suggests that the use of POCUS during cardiac arrest may have a direct impact on clinical outcomes. However, more randomized trials are required to establish the role of POCUS in cardiac arrest.

The same trend was also noticed for the interventions used, of which the rate of endotracheal intubation was significantly higher for patients with cardiac activity than those without and those who did not undergo POCUS exam (95.23% (95% CI 86.13–104.35) vs. 46.54% (95% CI 38.79–54.29) vs. 65.11% (95% CI 50.87–79.36), respectively; *p* < 0.001) [12]. The study also showed that epinephrine was given to a larger proportion of patients with cardiac activity than those without or those who did not undergo POCUS (100%; 100–100 vs. 82.39%; 76.5–88.3 vs. 81.39%; 69.76–93.03, respectively; < 0.001). Gaspari and colleagues also showed that the duration for resuscitation was longer when cardiac activity was recorded on the POCUS than when the POCUS showed cardiac standstill (18 min (IQR 10–30) vs. 12 min, (IQR 8–17), *p* < 0.05) [21]. However, this study reported no difference in the time between the doses of epinephrine. Similarly, a 2017 retrospective study reported that when patients with organized cardiac activity recorded in POCUS were treated with epinephrine, the ROSC and SHA rates were higher (54.7 and 37.7%). The study further reports that patients that recorded disorganized cardiac activity on POCUS and were treated with the standard ACLS interventions had significantly lower ROSC and SHA rates (37.2 and 17.9%, respectively; *p* < 0.005) [43].

## Limitations

The analysis made in the current study should be interpreted with consideration of the following weaknesses. First, it should be noted that the eligibility criteria of the present study allowed the inclusion of scientific journals published in English only, which might have introduced selection bias in our analysis. Additionally, the eligibility criteria only included studies from the year 2000 because we wanted to have more recent information on the prognostic value of POCUS; thus, other studies relevant to our topic may have been omitted, thus increasing the selection bias of our study. Secondly, all the studies included in this review were designed as observational studies meaning that the resuscitators were not blinded to the POCUS results. This might have influenced the decision of the resuscitators to stop resuscitation efforts after seeing cardiac standstill in the POCUS results. To minimize this bias, it is essential that all the resuscitative efforts are done for a specified period, irrespective of the POCUS results. Thirdly, for studies where cardiac activity was not defined, we opted assign them “unspecified” without contacting the authors meaning that our analysis may have had some reporting bias. Lastly, we could not perform any meta-analyses on clinical outcomes due to the high risk of bias and heterogeneity; therefore, only quantitative information was provided to analyze the role of POCUS in predicting resuscitation outcomes of cardiac arrest patients.

## Conclusion

POCUS has inconsistent prognostic value; hence, should not be used as the sole predictor in determining the termination of resuscitation efforts in cardiac arrest patients. Moreover, a more unified definition for cardiac activity is required to facilitate better interpretation of future outcomes. In addition, the level of POCUS training has no influence on the clinical outcomes. However, more high-quality randomized trials are required to support this finding.

## Data Availability

Not applicable.

## Appendix

See Table 2

**Table 2:** Prognostic test performance of POCUS findings stratified according to cardiac activity definition, initial cardiac rhythm and level of POCUS training

PICO	Subgroup	Outcomes	Author (year)	Sensitivity (95% CI)	Specificity (95% CI)
Does presence of cardiac activity on pre-hospital POCUS predict clinical outcomes during cardiac arrest	Initial cardiac arrest rhythm				
	PEA	SHA	Aichinger (2012)	1.00 (0.25–1.00)	0.70 (0.35–0.93)
			Breitkreutz (2010)	0.95 (0.77–1.00)	0.41 (0.24–0.61)
	Asystole	SHA	Aichinger (2012)	0.50 (0.13–0.99)	1.00 (0.81–1.00)
			Breitkreutz (2010)	0.69 (0.39–0.91)	0.83 (0.62–0.95)
	VF/VT	SHA	Aichinger (2012)	1.00 (0.15–1.00)	0.67 (0.30–0.93)
	Definition of cardiac activity				
	Unspecified cardiac activity	SHA	Breitkreutz (2010)	0.86 (0.70–0.94)	0.60 (0.47–0.725)
	Any movement of the myocardium	SHA	Aichinger (2012)	0.80 (0.31–0.97)	0.84 (0.68–0.93)
Does presence of cardiac activity on in-hospital (ICU & ED) POCUS predict clinical outcomes of cardiac arrest patients	Initial cardiac arrest rhythm				
	PEA	ROSC	Chardoli (2012)	1.00 (0.81–1.00)	0.33 (0.18–0.52)
			Kim (2016)	1.00 (0.54–1.00)	0.50 (0.01–0.99)
			Salen (2005)	1.00 (0.63–1.00)	0.88 (0.70–0.98)
			Tomruk (2012)	0.43 (0.26–0.61)	0.76 (0.56–0.90)
		SHA	Cureton (2012)	0.80 (0.28–0.99)	0.80 (0.69–0.89)
			Cebicci (2014)	0.93 (0.82–0.99)	0.93 (0.78–0.99)
			Blaivas (2001)	1.00 (0.74–1.00)	0.77 (0.56 – 0.91)
			Chua (2017)	0.67 (0.22–0.96)	0.88 (0.68–0.97)
			Salen (2001)	0.89 (0.52–1.00)	0.50 (0.35–0.65)
	Asystole	ROSC	Kim (2016)	0.0 (0.0–0.16)	1.00 (0.81–1.00)
			Salen (2005)	0.0	1.00 (0.90–1.00)
			Tomruk (2012)	0.11 (0.03–0.26)	0.97 (0.87–1.00)
		SHA	Cebicci (2014)	1.00 (0.15–1.00)	1.00 (0.98–1.00)
			Blaivas (2001)	0.0	1.00 (0.94–1.00)
			Chua (2017)	0.67 (0.30–0.93)	0.85 (0.72–0.94)
			Salen (2001)	0.0 (0.0–0.98)	0.91 (0.77–0.98)
	VF/VT	ROSC	Kim (2016)	1.00 (0.03–100)	0.0
			Tomruk (2012)	0.0 (0.0–0.60)	1.00 (0.40–1.00)
		SHA	Cebbici (2014)	0.94 (0.79–0.99)	0.62 (0.32–0.86)
			Blaivas (2001)	1.00 (0.63–1.00)	0.88 (0.76–0.95)
			Salen (2001)	1.00 (0.29–1.00)	0.50 (0.16–0.84)
	Definition of cardiac activity				
	Unspecified cardiac activity	ROSC	Lien (2018)	0.62 (0.50–0.73)	0.98 (0.95–100)
			Bolvardi (2016)	0.73 (0.60–0.84)	0.92 (0.85–0.96)
		SHA	Breitkreutz (2010)	0.86 (0.70–0.94)	0.60 (0.47–0.73)
			Cebbici (2014)	0.94 (0.86–0.97)	0.98 (0.95–0.99)
			Chua (2017)	0.72 (0.48–0.88)	0.84 (0.75–0.91)
		SHD	Lien (2018)	0.48 (0.28–0.69)	0.77 (0.69–0.83)
	Any movement of the myocardium	ROSC	Gaspari (2016)	0.64 (0.57–0.70)	0.78 (0.74–0.81)
			Thandar (2023)	0.52 (0.33–0.71)	0.95 (0.86–0.98)
		SHA	Aichinger (2012)	0.80 (0.31–0.97)	0.84 (0.68–0.93)
			Blaivas (2001)	0.98 (0.71–1.00)	0.91 (0.85–0.95)
			Gaspari (2016)	0.67 (0.58–0.75)	0.73 (0.69–0.76)
			Thandar (2023)	0.31 (0.13–0.57)	0.84 (0.77–0.89)
		SHD	Gaspari (2016)	0.77 (0.48–0.92)	0.68 (0.64–0.71)
			Thandar (2023)	0.50 (0.06–0.94)	0.83 (0.73–0.90)
			Zengin (2016)	0.40 (0.10–0.80)	0.77 (0.67–0.84)
	Any atrial, valvular or ventricular Movement	ROSC	Chardoli (2012)	1.00 (0.81–1.00)	0.33 (0.18–0.52)
			Hayhurst (2010)	0.92 (0.59–0.99)	0.76 (0.60–0.87)
			Kim (2016)	0.25 (0.11–0.45)	0.95 (0.75–1.00)
			Salen (2005)	0.94 (0.49–1.00)	0.94 (0.85–0.98)
			Masoumi (2021)	0.61 (0.44–0.75)	0.82 (0.74–0.88)
			Ozen (2016)	0.95 (0.85–0.98)	0.70 (0.58–0.80)
			Tomruk (2012)	0.26 (0.17–0.37)	0.89 (0.80–0.95)
		SHA	Hayhurst (2010)	0.80 (0.31–0.97)	0.64 (0.50–0.77)
			Masoumi (2021)	0.75 (0.52–0.89)	0.79 (0.71–0.85)
			Ozen (2016)	0.98 (0.86–1.00)	0.60 (0.49–0.70)
		SHD	Tayal (2003)	1.00 (0.59–1.00)	0.62 (0.32–0.86)
			Masoumi (2021)	0.85 (0.42–0.99)	0.74 (0.66–0.81)
	Organized cardiac activity	ROSC	Atkinson (2019)	0.34 (0.21–0.49)	0.96 (0.91–0.99)
			Flato (2015)	0.53 (0.35–0.70)	0.38 (0.13–0.68)
		SHA	Atkinson (2019)	0.39 (0.20–0.62)	0.91 (0.86–0.95)
			Cureton (2012)	0.86 (0.42–0.98)	0.91 (0.85–0.95)
		SHD	Atkinson (2019)	0.67 (0.09–0.99)	0.89 (0.84–0.93)
			Flato (2015)	1.00 (0.54–1.00)	0.51 (0.35–0.67)
Does level of POCUS training influence the ability to predict clinical outcomes of cardiac arrest	Inexperienced	ROSC	Lien (2018)	0.62 (0.50–0.73)	0.98 (0.95–100)
			Thandar (2023)	0.52 (0.33–0.71)	0.95 (0.86–0.98)
			Tomruk (2012)	0.26 (0.17–0.37)	0.89 (0.80–0.95)
		SHA	Aichinger (2012)	0.80 (0.31–0.97)	0.84 (0.68–0.93)
			Blaivas (2001)	0.98 (0.71–1.00)	0.91 (0.85–0.95)
			Chua (2012)	0.72 (0.48–0.88)	0.84 (0.75–0.91)
			Salen (2001)	0.85 (0.55–0.96)	0.67 (0.56–0.76)
			Thandar (2023)	0.31 (0.13–0.57)	0.84 (0.77–0.89)
		SHD	Lien (2018)	0.48 (0.28–0.69)	0.77 (0.69–0.83)
			Thandar (2023)	0.50 (0.06–0.94)	0.83 (0.73–0.90)
			Zengin (2016)	0.40 (0.10–0.80)	0.77 (0.67–0.84)
			Tayal (2003)	1.00 (0.59–1.00)	0.62 (0.32–0.86)
	Experienced	ROSC	Atkinson (2019)	0.34 (0.21–0.49)	0.96 (0.91–0.99)
			Masoumi (2021)	0.61 (0.44–0.75)	0.82 (0.74–0.88)
			Hayhurst (2010)	0.92 (0.59–0.99)	0.76 (0.60–0.87)
			Kim (2016)	0.25 (0.11–0.45)	0.95 (0.75–1.00)
			Ozen (2016)	0.95 (0.85–0.98)	0.70 (0.58–0.80)
			Gaspari (2016)	0.64 (0.57–0.70)	0.78 (0.74–0.81)
		SHA	Atkinson (2019)	0.39 (0.20–0.62)	0.91 (0.86–0.95)
			Cureton (2012)	0.86 (0.42–0.98)	0.91 (0.85–0.95)
			Masoumi (2021)	0.75 (0.52–0.89)	0.79 (0.71–0.85)
			Gaspari (2016)	0.67 (0.58–0.75)	0.73 (0.69–0.76)
			Hayhurst (2010)	0.80 (0.31–0.97)	0.64 (0.50–0.77)
			Cebbici (2014)	0.94 (0.86–0.97)	0.98 (0.95–0.99)
		SHD	Atkinson (2019)	0.67 (0.09–0.99)	0.89 (0.84–0.93)
			Masoumi (2021)	0.85 (0.42–0.99)	0.74 (0.66–0.81)
			Gaspari (2016)	0.77 (0.48–0.92)	0.68 (0.64–0.71)
			Flato (2015)	1.00 (0.54–1.00)	0.51 (0.35–0.67)
